# The Peri-Implant and Periodontal Microbiota in Patients with and without Clinical Signs of Inflammation

**DOI:** 10.3390/dj3020024

**Published:** 2015-03-31

**Authors:** Meike Luise Jakobi, Sascha Nico Stumpp, Meike Stiesch, Jörg Eberhard, Wieland Heuer

**Affiliations:** 1 Private Dental Practice, Hildesheim 31134, Germany; E-Mail: jakobi.meike@gmx.de; 2 Department of Prosthetic Dentistry and Biomedical Materials Science, Hannover Medical School, Carl-Neuberg-Strasse 1, Hannover 30625, Germany; E-Mails: stumpp.nico@mh-hannover.de (S.N.S.); stiesch.meike@mh-hannover.de (M.S.); eberhard.joerg@mh-hannover.de (J.E.)

**Keywords:** bacterial biofilms, dental implants, microbial diversity, single strand conformation polymorphism/SSCP, peri-implantitis

## Abstract

Late implant failures, caused by the inflammation of surrounding tissues are a problem in implant dentistry. The path of bacterial transmission from teeth to implants is not completely understood. Therefore, the purpose of this study was to analyze intraindividual bacterial transmission characterizing subgingival microbiomes in teeth and implants, both in healthy subjects and in those with signs of periodontitis or peri-implantitis. Samples of peri-implant and dental sulcus fluid were collected. To identify the predominant microbiota, amplified fragments of bacterial 16S rRNA gene were separated by single strand conformation polymorphism analysis, sequenced and taxonomically classified. A total of 25 different predominant genera were found in the diseased group and 14 genera in the healthy group. Species richness did not differ significantly between implants, neighboring teeth and teeth with largest probing depth in the diseased group. Additionally, no differences between teeth and implants in the healthy group were detected. In contrast, microbial diversity varied between the different sampling points. Species richness is similar in healthy and diseased sites, but the composition of the bacterial community differed within the individual subjects. The underlying analyses strongly suggest that complete transmission from neighboring teeth to implants is unlikely.

## 1. Introduction

Peri-implant diseases, such as peri-implantitis or mucositis, are a challenge in implant dentistry, as they are one of the main causes—besides implant loading conditions—of late implant failures [1]. With broad range molecular detection methods, more than 600 bacterial species have been identified that colonize different ecological niches in the human mouth [2]. Microorganisms populating surfaces are gradually organized into complex biofilms. Species within the biofilm interact specifically with each other. For example, early colonizers, such as *Streptococcus* or *Actinomyces* species, are essential for the attachment of late-colonizing gram-negative species [2,3,4]. Numerous studies have elucidated the pathogenic microbial processes leading from healthy to infected peri-implant tissues. Biofilm formation around implants is characterized by a shift from mainly gram-positive aerobic and facultative anaerobic cocci and rods to a higher proportion of periodontal pathogens [5,6,7,8,9]. According to Socransky *et al.* [10], this includes *Aggregatibacter actinomycetemcomitans*, as well as species of the red complex, such as *Porphyromonas gingivalis*, *Treponema denticola*, and *Tannerella forsythia,* and of the orange complex, such as *Fusobacterium nucleatum* and *Prevotella intermedia* [11,12].

Several studies have focused on periodontal pathogens and have demonstrated similarity between the microbiota around teeth and implants. It was, therefore, concluded that there is cross-contamination from teeth to implants [13,14,15,16,17,18]. These studies employed different techniques for the detection of potential pathogens, such as microbial culture, nucleic acid hybridization assays, and specific polymerase chain reactions (PCR). However, these methods target only predefined or cultivable bacterial phylotypes and are not able to determine the overall microbial diversity within the tested biofilms [19,20,21,22]. Therefore these detection techniques do not adequately identify potential differences in microbial composition in teeth or implants [23]. 16S ribosomal deoxyribonucleic acid (rDNA) broad range PCR amplification, in combination with single strand conformation polymorphism (SSCP) analysis, can non-specifically identify the predominant members within complex bacterial communities and has already been successfully used in numerous investigations exploring microbial diversity [2,24,25,26,27,28].

In contrast to studies that assumed transmission of bacteria from residual teeth to implants, recent studies have found evidence for differences in the colonization pattern. Some studies using DNA-DNA hybridization techniques showed differences in the specific bacterial species counts on teeth and implants in different phases of biofilm development [7,29,30]. Furthermore, other studies have found certain *Staphylococcus* species pluralis (spp.) and coliform bacteria around diseased implants that are not usually linked to periodontal infected sites [5,8,30,31,32]. Heuer *et al.* used a broad range technique and found differences in microbial diversity around teeth and implant sites with gingivitis or mucositis, respectively, so that they excluded complete transmission from infected teeth to implants [26]. This survey of Heuer *et al.* served as the starting-point to search for site-specific differences in the microbial composition of biofilms around teeth and implants in healthy patients and subjects who suffered from peri-implantitis or periodontitis by employing a broad range molecular detection method, to verify or reject the hypothesis that there is no difference in microbial diversity between implants and the remaining dentition. 

## 2. Material and Methods

### 2.1. Subjects

This study was authorized by the ethics committee of Hannover Medical School (No. 3791). Each patient was informed orally and in writing about the procedure and signed a consent form.

The analyses were based on nine subjects (partially edentulous, eight woman, one man, aged between 21 and 71 years (mean 53 ± 18 years)) with healthy peri-implant and mucosal tissues and nine subjects (partially edentulous, five woman, four men, aged between 42 and 71 years (mean 58 ± 9 years)) with signs of peri-implantitis and periodontitis. 

ll patients had at least one multipart titanium implant (Astra Tech, Mölndal, Sweden; Straumann, Basel, Switzerland) which was inserted in a single step operation between 2006 and 2009 in the upper or lower jaw and had been loaded three months after implant surgery at the earliest, with crowns or bridges cemented on an abutment.

To be included in the assessment, the cemented supra constructions had to be *in situ* for at least six months at the time of the sampling (August 2010–October 2010), the last professional dental cleaning had to date back at least three months and the subjects had to fulfill the following criteria: non-smoker, no systematic diseases like diabetes, rheumatic disease, osteoporosis, or leukemia, and no intake of antibiotics during or up to four months before sampling.

### 2.2. Periodontal and Peri-Implant Examination

The complete periodontal status—including the measurement of gingiva recession, pocket depth, plaque index and bleeding on probing (BOP)—was determined for each patient. The pocket depths and BOP were analyzed at six different sites on the tooth and the implant (mesio-buccal, buccal, disto-buccal, mesio-oral, oral, disto-oral). Subsequently to sampling, the plaque index (according to Silness and Loe) was measured at four sites per tooth (mesial, distal, oral, and vestibular).

To determine pocket depth, a marked periodontal probe was used for teeth (WHO-DMS probe, GY12 DMS, Deppeler SA, Rolle, Switzerland) and the PP12 DMS probe for implants (Deppeler SA, Rolle, Switzerland). As differences in probing pressure can produce different clinical results, all clinical examinations were carried out by the same trained clinician. The probing depth was measured to the nearest millimeter on the scale. Clinical data was compared using the Student’s t test. The general level of significance was set to p ≤ 0.05. Due to multiple testing, a Bonferroni correction was conducted and the level of significance was set to p ≤ 0.016 (*i.e*., 0.05/3), when three different sample sites were compared and to p ≤ 0.025 (*i.e*., 0.05/2), when two different sites were compared. 

### 2.3. Sampling

Samples were taken from periodontal healthy patients at an implant and its neighboring tooth. From patients with periodontal and peri-implant disease, samples were taken at the tooth with the greatest probing depth, the implant with the greatest probing depth and its neighboring tooth. According to the classifications of the American Academy of Periodontology [33], teeth were considered to exhibit periodontal disease when they presented signs of inflammation, such as redness, swelling, BOP, and probing depths above 3 mm. In this context, patients with pocket depths smaller than 3 mm were regarded as being free of periodontal disease. In order to obtain consistent results, the same procedure was followed for the implant sites, which is in accordance with the Consensus Report of the Seventh European Workshop on Periodontology [23], in which implants were regarded as diseased when they exhibited BOP, mobility or suppuration and as healthy when these signs were absent. The respective teeth and implants were dried with cotton rolls and by carefully removing the saliva film with an air spray. 

Four sterile paper points of size 35 were inserted at four points (mesio-vestibular, disto-vestibular, mesio-oral, and disto-oral) for ten seconds. Sterile forceps were used for each tooth and implant. Subsequently, the four paper points were pooled and stored in sterile 1.5 mL reaction vessels (Eppendorf AG, Hamburg, Germany) at −80 °C until further processing. Sampling and measurements were carried out by the same dentist.

### 2.4. DNA Isolation, Amplification of the 16S rDNA and Exonuclease Digestion

For the extraction of genomic DNA, the bacterial cells were mechanically disrupted using a bead mill (Precellys^®^24, Bertin Technologies, Montigny-le-Bretonneux, France). The total DNA was purified using the QIAmp DNA Mini Kit (Qiagen, Hilden, Germany), according to the manufacturer’s protocol for bacteria. Isolated DNA was stored at −20 °C until further processing. 

16S rDNA PCR amplification, as well as DNA pre-treatment for the SSCP analyses, were carried out as described by Heuer *et al.* [26]. 

### 2.5. SSCP Gel-Electrophoresis Separation of 16S rDNA Fragments According to Their Sequence

Single-strand conformation polymorphism (SSCP) analyses were carried out on a DCode Universal Mutation Detection System (Bio-Rad, Hercules, CA, USA), using 8% polyacrylamide gels (Bio-Rad). The electrophoresis was conducted at 360 V, at 20 °C for 24 hours in 1 x TBE buffer. 

### 2.6. Band Extraction, Re-Amplification, Sequencing

SSCP band profile was visualized by silver-staining according to the manufacturer’s protocol, (Silver-Stain Kit, Bio-Rad, Hercules, USA), followed by photographic documentation. The bands were cut out from the gel and the DNA was eluted overnight in elution buffer (0.5 M ammonium acetate, 10 mM magnesium acetate, 1 mM EDTA, 0.1% sodium dodecylsulfate, pH 8.0). Eluted DNA was concentrated and used as template for PCR re-amplification. Amplicons were purified using the MinElute PCR Purification Kit (Qiagen, Hilden, Germany) and subsequently sequenced by a commercial supplier (Seqlab, Göttingen, Germany). The sequences were analyzed using the BioEdit software package (v7.0.9, Ibis Biosciences, Carlsbad, CA, USA) and taxonomically classified by comparing similarity with the BLAST and RDB database sequences. 

For the classification to the species level, a minimum sequence similarity of 97% was chosen. Genus level identification was according to the RDB Classifier program with a predefined bootstrap cutoff value of 80%. Designation of as yet incompletely classified genus-level phylotypes was according to the Human Oral Microbiome Database [34].

### 2.7. Counting the SSCP Profiles and Statistical Analysis

The Quantity One 1D-Analysis Software package (v4.6.5, Bio-Rad) was used for the evaluation of the individual 16S rDNA banding patterns.

The statistical analysis compared the microbial diversity of sulcus fluid around implants to the remaining dentition and the null hypothesis is rejected if a significant difference is detected between implants and remaining dentition. 

The null hypothesis is:

-H0 _(1)_: No difference in microbial diversity between implants and the remaining dentition.
-HA_(1)_: Significant difference in microbial diversity between implants and the remaining dentition.

Comparison of the data was performed using a two-tailed Wilcoxon test for paired data. The level of significance was set to p ≤ 0.05.

Data documentation and evaluation was performed with the data processing program SPSS/PC Version 20.0 for Windows (SPSS, Chicago, IL, USA). 

The band migration patterns within individual patients were also compared. Bands occurring at the same height (±4%) within the gels were assigned as belonging to the same bacterial species (low diversity), whereas differences in the observed patterns are indicative of an altered microbial community composition (high diversity). 

## 3. Results

### 3.1. Clinical Examination

Comparison of the site-specific results in the healthy group and the group suffering from periodontitis/peri-implantitis demonstrated statistically significant differences for probing depth measurements and BOP values (Table 1).

Within the diseased group (Table 2), probing depth measurements were significantly different between the remaining complete dentition (3.6 mm ± 0.7 mm) and all implants (4.8 mm ± 0.5 mm), as well as between sampling point implants (5.2 mm ± 0.6 mm) and their neighboring teeth (3.9 mm ± 1.0 mm) No significant difference was found between the teeth with the greatest probing depth (5.6 mm ± 1.5 mm) and sampling point implants or between the teeth with the greatest probing depth and the neighboring teeth. BOP values did not differ significantly between the remaining dentition and all implants or between the specific sample sites.

In the healthy group (Table 3), the probing depth measurements did not differ significantly between the remaining dentition (2.4 mm ± 0.2 mm) and all implants (2.6 mm ± 0.4 mm) or between sampling point implants (2.8 ± 0.4) and their neighboring teeth (2.4 ± 0.5). The BOP results did not differ between all these sample sites.

**Table 1:** Comparison of plaque index, probing depth and bleeding on probing between the healthy and diseased group and corresponding p values.

Results at all remaining teeth and all implants in each subject																													
Sample site		Group	Plaque index	Corresponding p values								Probing depth [mm]	Corresponding p values								Bleeding on probing [%]	Corresponding p values							
Implants		Diseased group	0.6 ± 0.7	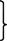	0.75	0.177		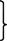	0.01			4.8 ± 0.5	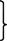	< 0.001	< 0.001		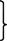	0.002			54.7 ± 18.2	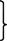	< 0.001	< 0.001		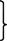	< 0.001		
		Healthy group	0.4 ± 0.7									2.6 ± 0.4									7.7 ± 6.6								
Teeth		Diseased group	1.6 ± 0.8	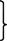	0.313							3.6 ± 0.7	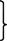	0.001							49.6 ± 19.7	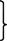	0						
		Healthy group	1.1 ± 0.9									2.4 ± 0.2									9.4 ± 6.1								
**Results at different sample sites in each subject**																													
Implants		Diseased group	0.7 ± 0.7	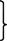	0.52	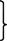	0.01	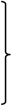	0.01	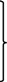	0.384	5.2 ± 0.6	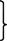	< 0.001	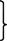	0.014	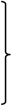	< 0.001	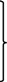	< 0.001	57.4 ± 19.4	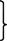	< 0.001	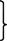	< 0.001	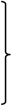	< 0.001	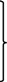	< 0.001
		Healthy group	0.4 ± 0.7									2.8 ± 0.4									5.6 ± 7.9								
Teeth	Neighboring teeth	Diseased group	1.6 ± 0.8	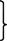	0.196							3.9 ± 1.0	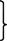	0.002							50.0 ± 15.7	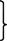	< 0.001						
	Neighboring teeth	Healthy group	1.0 ± 0.8									2.4 ± 0.5									7.4 ± 8.3								
	Teeth with greatest probing depth	Diseased group	1.6 ± 0.8	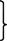	0.196							5.6 ± 1.5	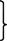	< 0.001							59.3 ± 11.4	< 0.001							
	Neighboring teeth	Healthy group	1.0 ± 0.8									2.4 ± 0.5									7.4 ± 8.3								

**Table 2:** Patients with signs of periodontitis/peri-implantitis: plaque index, probing depth and bleeding on probing at implant or tooth sites (mean and standard deviation) and corresponding p values.

Results at all remaining teeth and all implants in each subject																					
Sample site	Plaque index	Corresponding p values						Probing depth [mm]	Corresponding p values						Bleeding on probing [%]	Corresponding p values					
Remaining teeth	1.6 ± 0.8	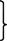	0.011					3.6 ± 0.7	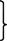	0.002					49.6 ± 19.7	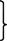	0.629				
Implants	0.6 ± 0.7							4.8 ± 0.5							54.7 ± 18.2						
**Results at different sample sites in each subject**																					
Teeth with greatest probing depth	1.6 ± 0.8	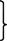	1	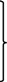	0.03			5.6 ± 1.5	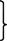	0.019	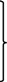	0.57			59.3 ± 11.4	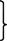	0.196	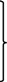	0.819	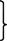	0.414
Neighboring teeth	1.6 ± 0.8					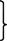	0.031	3.9 ± 1.0					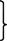	0.005	50.0 ± 15.7						
Implants	0.7 ± 0.7							5.2 ± 0.6							57.4 ± 19.4						

**Table 3:** Patients with healthy tissues around teeth and implants: plaque index, probing depth and bleeding on probing at implant or tooth sites (mean and standard deviation) and corresponding p values.

Results at all remaining teeth and all implants in each subject													
Sample site	Plaque index	Corresponding p values				Probing depth [mm]	Corresponding p values			Bleeding on probing [%]	Corresponding p values		
Remaining teeth	1.1 ± 0.9	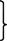	0.017			2.4 ± 0.2	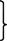	0.165		9.4 ± 6.1	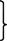	0.534	
Implants	0.4 ± 0.7					2.6 ± 0.4				7.7 ± 6.6			
Results at different sample sites in each subject													
Neighboring teeth	1.0 ± 0.8	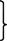	0.16			2.4 ± 0.5	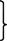	0.165		7.4 ± 8.3	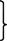	0.65	
Implants	0.4 ± 0.7					2.8 ± 0.4				5.6 ± 7.9			

### 3.2. Sequence-Dependent Separation of 16S rDNA Fragments 

For the evaluation of microbial diversity, the amplified bacterial 16S rDNA fragments were separated by SSCP. The 16S rDNA fragments with the same migration pattern in SSCP gel can be assigned to the same bacterial species.

In the healthy group, medial 6.2 ± 3.2 predominant bands per lane were found in the peri-implant sulcus and 5.9 ± 2.6 in the gingival sulcus of neighboring teeth. In the diseased group, medial 4.1 ± 2.7 predominant bands per lane were found in the peri-implant sulcus, 5.0 ± 1.8 in the gingival sulcus of neighboring teeth and 5.9 ± 3.7 in the gingival sulcus of teeth with the greatest probing depth. None of these differences were statistically significant. 

In 17 of 27 samples, comparison of individual band migration at different sampling sites found differences in microbial community composition at implant and tooth sites, both in the healthy, as well as in the diseased group. 

### 3.3. Sequence Analyses

Table 4 shows the total evaluation of the sequence analyses of the healthy and diseased group at the level of the genera and phylotypes. Table 5 shows the results on species-level or “species-level” phylotype in the diseased and healthy group at different sampling points.

#### 3.3.1. Diseased Group

In the diseased group, a total of 25 different predominant genera were found at all sites, of which 13 different genera were found at implants, 14 at neighboring teeth and 16 at teeth with greatest probing depth. The most frequent genera were *Enterococcus*, *Streptococcus*, *Porphyromonas, Fusobacterium Prevotella*, *Bacillus*, and *Fretibacterium.*

Members of the genera *Neisseria* and *Kingella* were exclusively found at implant sites, whereas *Fretibacterium* and unclassified bacilli were solely found at teeth sites (both neighboring teeth and teeth with greatest probing depth). The genera *Tannerella*, *Rothia*, *Parabacteroides*, *Parvimonas*, and *Filifactor* were only found at teeth sites with greatest probing depth but not at implants or their neighboring teeth. 

#### 3.3.2. Healthy Group

In the healthy group, a total of 14 different predominant genera were found, of which 10 different genera were found at teeth and 10 at implants. The most frequent genera were *Enterococcus*, *Bacillus*, *Streptococcus* and *Fusobacterium*. The genera *Veillonella*, *Capnocythophaga* and *Leptotrichia* were not found at implant sites, but isolated at tooth sites, in contrast to genera such as *Prevotella*, *Porphyromonas*, *Rothia* and *Proteus*, which were only found at implant sites. 

The results of the sequence analyses reject the null hypothesis of the study, that there is no difference in microbial diversity between implants and the remaining dentition.

**Table 4:** Bacterial phylotypes observed at different sampling points.

Phylotype	Patients with signs of periodontitis/peri-implantitis																														Patients with healthy tissues																		
	Implants										Neighboring teeth										Teeth with greatest probing depth										Implants										Neighboring teeth								
Patient No.	1	2	3	4	5	6	7	8	9		1	2	3	4	5	6	7	8	9		1	2	3	4	5	6	7	8	9		11	12	13	14	15	16	17	18	19		11	12	13	14	15	16	17	18	19
*Enterococcus*		•	•	•			•	•	•				•	•	•		•								•		•	•	•		•		•	•	•	•	•	•				•	•			•			•
*Streptococcus*	•			•	•	•	•					•		•		•	•		•				•	•							•	•							•		•	•							•
*Prevotella*							•				•	•	•	•		•								•								•																	
*Porphyromonas*					•	•						•		•		•					•	•		•	•	•						•																	
*Fusobacterium*	•						•						•	•	•							•		•		•						•						•			•			•				•	
*Bacillus*					•						•				•				•						•			•	•						•	•		•							•	•	•	•	
*Veillonella*					•		•																				•																						•
*Capnocytophaga*	•								•			•																													•								
*Paracoccus*				•									•																		•		•								•								•
*Fretibacterium*																•					•				•	•																							
*biotrophia*												•																																					
*Selenomonas*												•																																					
*Propionibacterium*											•																																						
*Peptostreptocooccus*														•																																			
*Tannerella*																													•																				
*Rothia*																												•											•										
*Parabacteroides*																													•																				
*Neisseria*	•																																																
*Kingella*	•																																																
*Leptotrichia*	•																										•														•	•							
*TM7* [G-1]																											•																						
*TM7* [G-5]							•																																										
*Parvimonas*																								•																									
*Filifactor*																										•																							
*Corynebacterium*																															•											•						•	
unclassified Clostridiales																																												•					
*Proteus*																																		•															
unclassified Bacilli																		•										•	•																				

**Table 5:** Results on species-level or species-level phylotype in the diseased and healthy groups at different sampling points.

Patients with periodontitis/peri-implantitis			
Patient No.		Genera/Phylotypes	Species/“Species-level” Phylotypes
**Implants**	1	*Neisseria*	*Neisseria* sp. oral taxon 014
		*Streptococcus*	*Streptococcus sanguinis*
		*Kingella*	
		*Leptotrichia*	*Leptotrichia* sp. oral taxon 213
		*Fusobacterium*	*Fusobacterium nucleatum*
		*Capnocytophaga*	*Capnocytophaga sputigena*
	2	*Enterococcus*	*Enterococcus* sp. oral taxon A43
	3	*Enterococcus*	*Enterococcus* sp. oral taxon A43
	4	*Enterococcus*	*Enterococcus* sp. oral taxon A43
		*Streptococcus*	*Streptococcus sanguinis*
		*Paracoccus*	*Paracoccus* sp. K1-202
	5	*Veillonella*	*Veillonella parvula*
		*Bacillus*	*Bacillus* sp. oral taxon B77
		*Streptococcus*	*Streptococcus mitis*
			*Streptococcus anginosus*
		*Porphyromonas*	*Porphyromonas gingivalis*
	6	*Streptococcus*	*Streptococcus* sp. oral taxon C08
		*Porphyromonas*	*Porphyromonas gingivalis*
	7	*Prevotella*	*Prevotella histicola*
		*Veillonella*	
		*Enterococcus*	*Enterococcus italicus*
		*Fusobacterium*	*Fusobacterium nucleatum*
		*Streptococcus*	
		*TM7* [G-5]	*TM7* [G-5] sp. oral taxon 437
	8	*Enterococcus*	*Enterococcus casseliflavus*
		*Bacillus*	*Bacillus cellulosilyticus*
		unclassified Bacilli	*Bacilli bacterium*oral taxon C43
	9	*Enterococcus*	
		*Capnocytophaga*	
**Neighboring teeth**	1	*Propionibacterium*	*Propionibacterium* sp. oral taxon 194
		*Prevotella*	
		*Prevotella*	*Prevotella* sp. oral taxon 303
		*Bacillus*	
	2	*Prevotella*	*Prevotella* sp. oral taxon 317
			*Prevotella nigrescens*
		*Selenomonas*	*Selenomonas artemidis*
		*biotrophia*	*biotrophia defectiva*
		*Streptococcus*	*Streptococcus sanguinis*
		*Porphyromonas*	*Porphyromonas gingivalis*
		*Capnocytophaga*	*Capnocytophaga* sp. oral taxon 329
	3	*Prevotella*	*Prevotella intermedia*
		*Enterococcus*	*Enterococcus casseliflavus*
			*Enterococcus* sp. oral taxon A43
		*Fusobacterium*	*Fusobacterium nucleatum*
			*Fusobacterium* sp. oral taxon C10
		*Paracoccus*	*Paracoccus* sp. K1-202
	4	*Prevotella*	*Prevotella* sp. oral taxon 472
		*Peptostreptocooccus*	*Peptostreptocooccus* sp. oral clone FG014
		*Enterococcus*	*Enterococcus* sp. oral taxon A43
		*Streptococcus*	*Streptococcus constellatus*
		*Fusobacterium*	*Fusobacterium nucleatum*
			*Fusobacterium* sp. oral taxon 203
		*Porphyromonas*	*Porphyromonas gingivalis*
	5	*Bacillus*	*Bacillus* sp. oral taxon B77
		*Enterococcus*	*Enterococcus* sp. oral taxon A43
		*Fusobacterium*	*Fusobacterium nucleatum*
	6	*Prevotella*	*Prevotella* sp. oral taxon 317
		*Streptococcus*	*Streptococcus cristatus*
		*Porphyromonas*	*Porphyromonas gingivalis*
		*Fretibacterium*	*Fretibacterium* sp. oral taxon 360
	7	*Enterococcus*	*Enterococcus* sp. oral taxon A78
			*Enterococcus italicus*
		*Streptococcus*	*Streptococcus sanguinis*
	8	unclassified Bacilli	*Bacilli bacterium*oral taxon C43
	9	*Bacillus*	*Bacillus* sp. oral taxon C44
		*Streptococcus*	*Streptococcus mitis*
**Teeth with greatest probing depth**	1	*Porphyromonas*	*Porphyromonas gingivalis*
		*Fretibacterium*	*Fretibacterium* sp.oral taxon 359
	2	*Fusobacterium*	*Fusobacterium nucleatum*
		*Porphyromonas*	*Porphyromonas gingivalis*
	3	*Streptococcus*	*Streptococcus mitis*
	4	*Prevotella*	*Prevotella* sp. oral taxon 472
			*Prevotella veroralis*
			*Prevotella* sp. oral taxon 306
			*Prevotella nigrescens*
		*Parvimonas*	
		*Streptococcus*	*Streptococcus constellatus*
		*Fusobacterium*	*Fusobacterium nucleatum*
			*Fusobacterium* sp. oral taxon 203
		*Porphyromonas*	*Porphyromonas* sp. oral clone BP1-92
			*Porphyromonas gingivalis*
	5	*Bacillus*	*Bacillus* sp. oral taxon B77
		*Enterococcus*	*Enterococcus* sp. oral taxon A43
		*Porphyromonas*	*Porphyromonas gingivalis*
		*Fretibacterium*	*Fretibacterium* sp. oral taxon 360
	6	*Filifactor*	*Filifactor alocis*
		*Fusobacterium*	*Fusobacterium* sp. oral taxon A11
		*Porphyromonas*	*Porphyromonas gingivalis*
		*Fretibacterium*	*Fretibacterium* sp. oral taxon 362
	7	*Veillonella*	
		*Enterococcus*	*Enterococcus italicus*
		*TM7* [G-1]	*TM7* [G-1] sp. oral taxon 347
		*Leptotrichia*	*Leptotrichia wadei*
	8	unclassified Bacilli	*Bacilli bacterium*oral taxon C43
		*Enterococcus*	*Enterococcus casseliflavus*
			*Enterococcus gallinarum*
		*Bacillus*	*Bacillus cellulosilyticus*
		*Rothia*	*Rothia dentocariosa*
	9	unclassified Bacilli	*Bacilli bacterium* oral taxon C43
		*Enterococcus*	*Enterococcus casseliflavus*
			*Enterococcus* sp. oral taxon A43
		*Bacillus*	*Bacillus cellulosilyticus*
		*Tannerella*	*Tannerella forsythia*
		*Parabacteroides*	
**Patients with healthy tissues**			
**Patient No.**		**Genera/Phylotypes**	**Species/“Species-level” Phylotypes**
**Implants**	11	*Paracoccus*	*Paracoccus* sp. K1-202
		*Corynebacterium*	*Corynebacterium matruchotii*
		*Streptococcus*	*Streptococcus oralis*
		*Enterococcus*	*Enterococcus faecalis*
	12	*Prevotella*	*Prevotella* sp. oral taxon 303
			*Prevotella melaninogenica*
			*Prevotella nigrescens*
		*Streptococcus*	*Streptococcus mitis*
		*Fusobacterium*	*Fusobacterium nucleatum*
			*Fusobacterium* sp. oral taxon C10
		*Porphyromonas*	*Porphyromonas endodontalis*
	13	*Enterococcus*	*Enterococcus* sp. oral taxon A43
		*Paracoccus*	*Paracoccus denitrificans*
			*Paracoccus* sp. TDMA-10
	14	*Enterococcus*	*Enterococcus gallinarum*
			*Enterococcus casseliflavus*
			*Enterococcus* sp. oral taxon A43
		*Proteus*	*Proteus* sp. oral taxon C50
	15	*Bacillus*	*Bacillus* sp. oral taxon B77
		*Enterococcus*	*Enterococcus* sp. oral taxon A43
	16	*Bacillus*	*Bacillus* sp. oral taxon B77
		*Enterococcus*	*Enterococcus* sp. oral taxon A43
	17	*Enterococcus*	
	18	*Bacillus*	*Bacillus* sp. oral taxon B77
		*Enterococcus*	*Enterococcus* sp. oral taxon A43
		*Fusobacterium*	*Fusobacterium nucleatum*
	19	*Rothia*	
		*Streptococcus*	
**Neighboring teeth**	11	*Streptococcus*	*Streptococcus mitis*
			*Streptococcus cristatus*
			*Streptococcus* sp. oral taxon E12
			*Streptococcus sanguinis*
		*Paracoccus*	*Paracoccus* sp. K1-202
		*Fusobacterium*	*Fusobacterium nucleatum*
		*Leptotrichia*	uncultured L *eptotrichia* sp.
		*Capnocytophaga*	*Capnocytophaga* sp. oral taxon 329
	12	*Corynebacterium*	*Corynebacterium matruchotii*
		*Enterococcus*	*Enterococcus faecalis*
		*Streptococcus*	*Streptococcus oralis*
			*Streptococcus mitis*
			*Streptococcus cristatus*
		*Leptotrichia*	*Leptotrichia buccalis*
	13	*Enterococcus*	*Enterococcus* sp. oral taxon A43
	14	unclassified Clostridiales	*Clostridiales bacterium* oral taxon C07
		*Fusobacterium*	*Fusobacterium* sp. oral taxon 203
	15	*Bacillus*	*Bacillus* sp. oral taxon B77
	16	*Bacillus*	*Bacillus* sp. oral taxon B77
		*Enterococcus*	*Enterococcus* sp. oral taxon A43
	17	*Bacillus*	*Bacillus* sp. oral taxon C44
			*Bacillus* sp. oral taxon B77
	18	*Corynebacterium*	*Corynebacterium matruchotii*
		*Bacillus*	*Bacillus* sp. oral taxon B77
		*Fusobacterium*	
	19	*Veilonella*	*Veillonella parvula*
		*Streptococcus*	*Streptococcus mutans*
		*Enterococcus*	
		*Paracoccus*	

## 4. Discussion

In this investigation, the microbial diversity of dental and implant habitats both in healthy subjects and in subjects with periodontitis/peri-implantitis was analyzed using a 16S rDNA-based SSCP approach, which allows accurate and sensitive sequence-dependent separation of 16S rDNA molecules.

In the two groups of study-subjects (healthy and infected with periodontitis/peri-implantitis), species richness was found to be similar in all tested habitats around teeth and implants. This observation was different to results presented by Heuer *et al.* [26], who found a significantly higher diversity around teeth with gingivitis than around implants with mucositis. This discrepancy may be, in part, explained through the more severe inflammation of the tissues in the present study. Diverse species richness in different oral habitats was also described by Kumar *et al.* [35], who demonstrated significantly higher diversity around teeth than around implants, both in health and disease. These results may be attributed to different probe sampling procedures. While Kumar *et al.* [35] pooled all samples from different dental sites within one subject, our analyses considered single site results within each individual. 

Previous studies suggested that peri-implant microbiota does not differ significantly from dental sulcus microbiota, neither in health nor in disease, and concluded thereof crossinfection of implant habitants by bacterial transmission [13,14,15,16,17,18]. Our results do not support this hypothesis. In our investigation, there are distinctively less microbial similarities between the different sampling sites both in health and disease. Of the great number of bacteria detected by our analyses, only the species *Porphyromonas gingivalis*, *Enterococcus italicus*, *Bacillus* sp. oral taxon B77, and *Bacilli bacterium* oral taxon C43 were present at all different sampling sites in single subjects of the diseased group. In one subject*, Enterococcus sp.* oral taxon A43 was present at the diseased implant, as well as at its adjacent tooth. In addition, in one subject each, *Enterococcus casseliflavus*, *Porphyromonas gingivalis*, *Bacillus cellulosilyticus*, and *Veillonella spp* were detected both at the implant and at the tooth with greatest probing depth. 

The presence of *Porphyromonas gingivalis* is in agreement with several investigations, which have found this typical member of the red complex according to Socransky *et al.* [10] in an increased number in cases of peri-implantitis [9,36,37]. However, these studies also found *ggregatibacter actinomycetemcomitans* as well as species from the red complex, such as *Treponema denticola* and *Tannerella forsythia*, and orange complex species, such as *Fusobacterium nucleatum* and *Prevotella intermedia* [11,12], in an increased number in cases of disease. The incidence of these other red and orange complex species is not proved by our observations. *Treponema denticola* or *Aggregatibacter actinomycetemcomitans* were never detected in our peri-implantitis group and *Tannerella forsythia* on only one occasion. Our findings are supported by the investigations of Koyanagi *et al.* and Renvert *et al.* [27,38] who also found only low levels of periodontopathic bacteria, such as *Porphyromonas gingivalis*, in peri-implant lesions. They are also in agreement with other studies that have noted that subjects with peri-implantitis or failing implants do not always exhibit periodontopathic bacteria [16,39,40].

Numerous investigations have reported a relationship between peri-implantitis and the occurrence of enteric rods [5,8,29,30,41]. Charalampakis *et al.* [42] found an increased number of enteric rods with a prevalence of 18.6% in a group of 281 patients. *Enterococci* are generally considered as increasingly important community-acquired and nosocomial pathogens. Even if they are regarded as to be of a low pathogenic potential, they can cause serious invasive infections, such as endocarditis, urinary tract-, pelvic-, and intra-abdominal infections, and bacteremia [43]. Investigations have shown that *Enterococcus casseliflavus* populates the gastrointestinal tract of both healthy and hospitalized persons and that it is a common part of the stool flora of the general population [44]. *Enterococcus italicus* is an enterococcal species widely diffused in dairy products [45]. It is also possible that its appearance could be linked to nutrimental factors. The role of *Enterococci* in biofilm formation around implants and its impact for pathogenic processes need further investigations.

*Bacillus cellulosilyticus* has been described as one of alkaliphilic bacterial strains which have important impact in industrial applications or enzyme studies due to their ability in producing alkaline and extracellular enzymes that are resistant to high pH and/or high temperature conditions [46]. But until now, for this bacterium, as well as for *Bacillus* sp. oral taxon B77 or *Bacilli bacterium* oral taxon C43, no pathogenic role in peri-implantitis development was described in any investigation.

In the healthy group, only *Enterococcus* sp. oral taxon A43, *Streptococcus mitis, Bacillus* sp. oral taxon B77 and *Paracoccus* sp. K1-202 were detected both at implants and adjacent teeth.

On the basis of the present investigation and data published by Preza *et al.* 2009 [47] and Dabdoub *et al.* [28], we conclude that the bacterial biofilm composition around teeth and implants is likely to be specific to the sampling site and that these sites could constitute distinct ecosystems. 

The used 16S rDNA-based SSCP method is a powerful tool to characterize complex microbial communities in terms of bacterial diversity and taxonomic assignment [48], thus simple culture fails to reproduce the real *in situ* diversity. Compared to other culture-independent molecular biological detection methods [49], such as specific PCR [50] or DNA-DNA hybridization [47,51], in which only anticipated bacteria can be tracked, the SSCP approach is not species-specific and covers ideally all bacteria present in a given sample. In the last few years, next generation sequencing techniques like Illumina sequencing or Pyrosequencing have also become interesting tools for microbial diversity analyses [52,53]. Nevertheless, sequencing costs are high and the data require massive computing power for processing and evaluation. In this regard, the SSCP is still the method of choice in the standard laboratory when high discriminatory power for microbial diversity analyses is needed.

The microbial fingerprintings including the SSCP technique have already been successfully applied in several related studies [24,25,26,54,55], however, the number of studies including patients suffering from periodontitis or peri-implantitis were limited [27,28,35]. To the best of our knowledge, the present investigation is the first utilizing a DNA fingerprinting technique for the evaluation of microbial diversities at inflamed implants, one of their adjacent teeth and an additional distant tooth with greatest probing depth in the same patient but further investigations are needed to define the role of different habitats. *In vitro* analyses have already indicated that implant surface texture and composition may affect peri-implant microbiomes, although the magnitude of this effect is still controversial [25].

## 5. Conclusions

The present study shows that the microbiome around implants does not exhibit greater biodiversity than teeth in the same subject. However, it seems that in each individual, microbial diversity around implants and teeth is different. Implants with signs of peri-implantitis do not always harbor typical periodontal pathogens. Thus, the investigation of polymicrobial diseases such as periodontitis and peri-implantitis should, not only focus on the typical periodontopathic bacteria, but also consider highly diverse biofilms and interactions between the different members within. 

Identification of the individual members within biofilms in healthy individuals or in patients with peri-implant infection is potentially of great significance in the development of preventive or therapeutic strategies.
